# Clinical outcomes associated with dynamic changes in serum sodium amongst adult patients with spontaneous subarachnoid hemorrhage and other critical illnesses: an exploratory scoping review

**DOI:** 10.62675/2965-2774.20260019

**Published:** 2026-07-07

**Authors:** Vignesh Raman, Mahesh Ramanan, Felicity Edwards, Zemedu Aweke Ferede, Vivienne Tippett, Kevin B. Laupland

**Affiliations:** 1 Queensland University of Technology Faculty of Health Brisbane Queensland Australia Faculty of Health, Queensland University of Technology - Brisbane, Queensland, Australia.

**Keywords:** Subarachnoid hemorrhage, Traumatic brain injury, Critical illness, Dysnatremia, Serum sodium, Intensive care units

## Abstract

Incident dysnatremia has been reported amongst varied intensive care unit disease populations and is associated with worse clinical outcomes, but less is known about the impacts of dynamic changes in serum sodium during intensive care unit admission. The primary objective of this study was to conduct a scoping review of the published literature on patient outcomes associated with dynamic changes in serum sodium in critically ill adults, to inform future research priorities. A scoping review was conducted according to the Joanna Briggs Institute method. PubMed^®^, Embase, CINAHL, Scopus, and Web of Science databases were searched for relevant articles on sodium "change", "trajectory", "fluctuation", or "variability" in adult patients managed in the intensive care unit. Seventeen articles were extracted; seven involved patients with subarachnoid hemorrhage in the intensive care unit, and ten involved mixed or other specific primary diagnoses requiring intensive care unit admission. In subarachnoid hemorrhage, higher magnitudes of serum sodium change, independent of incident dysnatremia, are associated with greater mortality and delayed cerebral ischemia. A similar association between the magnitude of serum sodium change and mortality is observed in other intensive care unit disease populations. There is limited literature on dynamic changes in serum sodium in intensive care unit populations, but current evidence suggests that greater magnitude is associated with higher mortality and morbidity across multiple intensive care unit disease populations. Current observational literature is insufficient to establish causal links between dysnatremia and worse patient outcomes.

## INTRODUCTION

Sodium, the predominant extracellular cation, is the major determinant of serum tonicity and intracellular volume and has a pivotal role in regulating neuronal cell excitation.^(1)^ Dysnatremias, resulting from impaired sodium regulation and/or water balance, are the commonest electrolyte disorders encountered in the intensive care unit (ICU).^(2,3)^ Dysnatremias can be further categorized into hyponatremia (serum sodium < 135mmol/L) or hypernatremia (serum sodium > 145mmol/L), both of which are more often acquired during ICU stay than pre-existing at the time of admission.^(3)^ Amongst selected ICU patient populations, hyponatremia has been reported in up to 38 % of cases, whilst hypernatremia was observed in up to 26% of cases.^(4,5)^

Both categories of incident dysnatremia have been associated with increased mortality, morbidity, and prolonged ICU stay,^(6)^ warranting early recognition and prompt treatment. However, one can appreciate that dynamic changes in serum sodium, caused both by dysnatramia treatment and as part of the patient's critical illness trajectory, can have potential detrimental effects. Dynamic changes in serum sodium may reflect compartmental fluid shifts, impacting hemodynamic parameters and neuronal recovery- both particularly problematic in the context of central nervous system (CNS) pathology.

There is growing recognition that dynamic changes in serum sodium in certain critical illnesses can be an independent predictor of patient mortality and morbidity, regardless of the patient's dysnatremia category.^(7-10)^ As such, the change in serum sodium, the magnitude or rate of change, or whether the absolute level of sodium concentration may moderate the effect of the change on serum sodium concentration, warrants more research focus. Further, it is unclear whether targeting a certain serum sodium value is associated with an improved outcome; this would be particularly invaluable to establish in certain disease categories encountered in the ICU, such as traumatic brain injury (TBI) and subarachnoid hemorrhage (SAH), where a focus on correcting abnormal serum sodium value is recommended to minimize secondary brain injury.^(11)^

The primary objective of this study was to conduct a scoping review of the published literature on patient outcomes associated with dynamic changes in serum sodium in critically ill adults, to inform future research priorities.

## METHODS

### Protocol design

The scoping review was reported in accordance with the Joanna Briggs Institute method^(12)^ and follows a five-stage methodological framework: establish research questions, screen for relevant studies, select studies, chart data, and summarise and report results.

### Research question

The primary research question was "What is the association between dynamic changes in serum sodium and patient outcomes in the ICU setting?"

The secondary research questions included: "Which disease processes have this association been studied in?"; "What particular clinical outcomes were investigated?"; and "What was the level of association reported?"

### Eligibility criteria

Study eligibility incorporated the Population, Concept, and Context mnemonic^(12)^ to identify primary studies and review articles focusing on patients in the adult ICU AND serum sodium "trajectory", "change", "fluctuation", or ‘variability".

Studies were excluded if they were: not published in English; including patients aged < 18 year; not involving human subjects; case reports; review articles and editorials that did not provide novel content; studies that did not investigate clinical patient outcomes; studies that included non-ICU populations; not accessible in a portable document format.

### Search strategy

The proposed search strategy was designed in consultation with an academic librarian. Five international electronic databases were searched; PubMed^®^ (Ovid, 1946- search date), Embase (Ovid, 1947- search date), Cumulative Index to Nursing and Allied Health Literature (CINAHL) (EBSCO host, 1984- search date), Scopus (EBSCO host, 1969- search date) and Web of Science (Clarivate, 1997- search date) using the key concepts "intensive care unit", "critical", "dysnatremia", "hyponatraemia", "hypernatraemia", "sodium". The databases had been selected to allow adequate coverage of primary and secondary publications with ICU, endocrinology, neurosurgery, neurology, and chemical pathology literature focuses. Searches on these databases were conducted between 1^st^ - 30^th^ March 2025 (Table 1S - Supplementary Material).

"Grey literature" was accessed by search parameters on the databases, including published preprints, university repositories, conference papers, and dissertation files.

### Study selection and quality assessment

Retrieved articles were exported and stored in EndNote^®^ 20 bibliographic and reference manager (Clarivate, 2013). After deduplication, a two-stage screening process was utilized using a commercially available data extraction tool (Covidence, 2023). The screening of titles and abstracts (stage 1) was performed independently by two reviewers, followed by full-text screening (stage 2) of publications deemed eligible and those in which the title or abstract provided insufficient information on eligibility. The senior author resolved uncertainty about study inclusion. Literature was graded on methodological quality according to the National Health and Medical Research Council (NHMRC) Evidence Hierarchy system.^(13)^

### Data extraction and reporting

A customized data extraction form was developed by one author using Microsoft^®^ Excel (Microsoft Corporation, Redmond, Washington, 2023) for manual data extraction by two authors. Extracted data included: first author, publication year, country of study, study design, study population, primary and secondary outcomes, and key findings.

**Figure 1:**
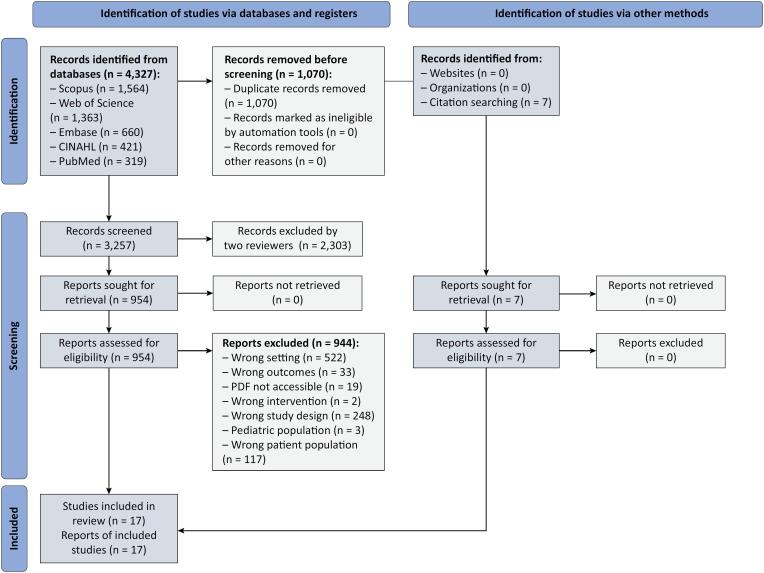
PRISMA flow diagram describing article extraction methodology.

Results were summarised in three ways: a Preferred Reporting Items for Systematic Reviews and Meta-Analysis (PRISMA) flow diagram (Figure 1) to present the study selection process; tables and figures to present data extracted from the eligible papers; a narrative summary describing the studies in relation to the objective and review questions.

## RESULTS

### Study selection

As summarized in figure 1, the database hit search process, the inclusion of results from citation searches, and the removal of duplicates resulted in 3,257 articles reaching the abstract and title screening stage. A total of 2,303 articles were excluded, and the remaining 954 articles were reviewed as full texts. At this stage, a further 944 articles met exclusion criteria and were excluded. A further seven articles were found through citation searching. A total of seventeen articles were included for final analysis.^(7-10,14-25)^

### Methodological quality

All included articles were observational study designs. No studies were randomized. Thirteen studies were retrospective designs^(8,10,14-19,21-23,25)^ and frequently reported vast heterogeneity in patient population and disease severity.^(17,18,21,24,22,26)^ All included studies were graded as NHMRC level III as they were comparative type cohort studies without concurrent controls.^(13)^

### Study characteristics and setting

Details of the 17 included studies are listed in tables 1 and 2. The studies varied vastly in population size; 2 studies had 1 - 199 participants,^(9,14)^ 6 studies had 200 - 500 participants,^(7,8,10,15,19,23)^ 2 with 501 - 1,000 participants^(20)^ and 7 studies that investigated multi-center cohorts between 1,001 - 37,000 participants.^(16-18,21,22,24,25)^

**Table 1:** Dynamic serum sodium change studies in patients with subarachnoid hemorrhage requiring intensive care unit management. Description of study characteristics and key findings

Authors	Study type	Country	Study size	Primary outcome	Secondary outcomes	Key findings
Cohen et al.^(7)^	Multi-center, prospective observational	Australia/ New Zealand	356	mRS	ICU LOS, hospital LOS	Greater variability in sodium concentrations was associated with lower mRS at 6 months, longer ICU and hospital length of stay
Eagles et al.^(8)^	Multi-center, retrospective observational	United States	413	DCI	N/A	There was a trend toward significance for the average absolute daily difference in sodium levels from admission levels during the vasospasm window as an independent predictor of DCI (p = 0.052). There was no difference in the predictive capacity for DCI when sodium fluctuations from post-aSAH days 1 - 14 were compared with those from the classic vasospasm window (days 3 - 12)
Harada et al.^(9)^	Multi-center, prospective observational	Japan	133	SVS	Stress-related hormone dynamics.	Serum sodium fluctuations were associated with SVS occurrence. Serum sodium fluctuations were associated with stress-related hormonal dynamics
Bales et al.^(14)^	Single-center, retrospective observational	United States	198	DCI	mRS	More patients with a sodium variability of 6 - 12 and > 12mEq/L had cerebral infarction than those with variability < 6mEq/L
Chua et al.^(15)^	Single-center, retrospective observational	United States	271	mRS	DCI, Radiological vasospasm, Mortality	There were no significant differences in inter-individual serum sodium values over time or in the occurrence of radiographic vasospasm, neurologic deterioration, functional outcomes, or mortality outcomes using the mixed-effect regression model. However, overall mean serum sodium levels were significantly higher in patients who had neurologic deterioration, poor functional outcome (mRS 3 - 6), and mortality
Jin et al.^(19)^	Multi-center, retrospective observational	United States	295	Hospital Mortality	N/A	Sodium fluctuations above 8.5mmol/L were independently associated with in-hospital mortality
Labib et al.^(20)^	Single-center, prospective observational	Netherlands	964	DCI	N/A	Hyponatremia, hypernatremia, and fluctuations of sodium levels were not predictive of DCI. Higher sodium levels, hypernatremia, and greater fluctuations of sodium levels were significantly associated with poor outcome in were significantly associated with poor outcome in both DCI and non-DCI patients

mRS - modified Rankin score; ICU - intensive care unit; LOS - length of stay; DCI - delayed cerebral ischemia; N/A - not applicable; aSAH - aneurysmal subarachnoid hemorrhage; SVS - symptomatic vasospasm.

**Table 2:** Dynamic serum sodium change studies in patients with other diagnoses requiring intensive care unit management. Description of study characteristics and key findings

Authors	Study type	Country	Study size, Primary diagnosis	Primary outcome	Secondary outcomes	Key findings
Darmon et al.^(16)^	Multi-center, prospective observational	France	11,125, unselected ICU patients	30-day mortality	N/A	Amongst unselected ICU patients, dysnatremia, including mild changes in serum sodium concentration, is an independent risk factor for 30-day mortality
Grim et al.^(17)^	Multi-center, retrospective observational	Netherlands	36,660, unselected ICU patients	Hospital mortality	N/A	Among unselected ICU patients, an increase in serum sodium within the first 48 hours of ICU admission was associated with higher in-hospital mortality in both normonatremic and hypernatremic patients
Huang et al.^(18)^	Single center, retrospective observational	United States	9,314, patients in the ICU with AKI	30-day mortality	1-year mortality	In patients with AKI, the serum sodium trajectories were independently associated with 30-day and 1-year mortality.
Marshall et al.^(21)^	Single center, retrospective observational	United States	8,600, surgical ICU patients	28-day mortality	1-year mortality, ICU mortality, and hospital mortality.	A significant association was found between sodium fluctuation and 28-day mortality, even in surgical patients who remained normonatremic during their ICU stay
Sakr et al.^(22)^	Single center, retrospective observational	Germany	10,923, surgical ICU patients	In-hospital mortality	N/A	A significant association was found between sodium fluctuation and in-hospital mortality, even in surgical patients who remained normonatremic during their ICU stay
Sen et al.^(23)^	Single center, retrospective observational	United States	212, severe burns	In-hospital mortality	N/A	After adjusting for total body surface area, age, ventilator days, and ICU length of stay, a higher coefficient of variation in sodium measurements was associated with mortality. Further, a large variation in sodium levels in the first 10 days of admission may be associated with increased mortality
Troché et al.^(24)^	Single-center, retrospective observational	France	215, patients in the ICU requiring RRT	Hospital mortality	ICU mortality	Among ICU patients requiring RRT, higher serum sodium was associated with increased mortality. The only significant variable associated with RRT-induced sodium change was dialysis sodium gradient
Shen et al.^(25)^	Single-center, retrospective observational	United States	1,038, patients in the ICU with sepsis after cardiac surgery	30-day mortality	N/A	Although the fluctuation in serum sodium remained within the normal range after cardiac surgery, a higher trajectory of serum sodium substantially increased the 30-day mortality risk in this patient cohort
Li et al.^(27)^	Single-center, retrospective observational	United States	514, patients in the ICU with sepsis and lactic acidosis	30-day mortality	N/A	Among patients with lactic acidosis complicated by sepsis, those with stable, normal fluctuations in serum sodium levels had better 30-day survival
Harrois et al.^(40)^	Multi-center, retrospective observational	Australia, Europe	240, severe TBI	28-day mortality	ICU LOS	After adjusting for baseline TBI severity, diabetes insipidus, the use of osmotherapy, the occurrence of hypernatremia hyponatremia, and center, daily serum sodium variability was significantly and independently associated with 28-day mortality

ICU - intensive care unit; N/A - not applicable; AKI - acute kidney injury; RRT - renal replacement therapy; TBI - traumatic brain injury; LOS - length of stay.

The studies were set across at least six countries, with two studies including patient data from multiple countries.^(7,10)^ Nine studies incorporated patient data from the United States,^(8,14,15,18,19,21,23,25,27)^ two from France,^(16,24)^ two from the Netherlands,^(17,20)^ one from Germany,^(22)^ one from Japan,^(9)^ one from Europe and Australia,^(10)^ and one from Australia and New Zealand.^(7)^

Seven of the studies focused primarily on patients admitted to the ICU after sustaining a spontaneous SAH,^(7-9,14,15,19,20)^ whilst the remainder were in other disease populations. Two of these studies were in mixed ICU patients,^(17,26)^ two in surgical ICU patients,^(21,22)^ two in acute renal dialysis patients,^(18,24)^ two in sepsis,^(25,27)^ one in TBI patients^(10)^ and one in burns.^(23)^ The authors further grouped these studies into themes of SAH populations or other ICU populations and summarized their findings below.

### Definitions of dynamic serum sodium change

Table 3 summarizes the terminology, definitions, and statistical techniques used to quantify dynamic changes in serum sodium. Three authors referred to group-based trajectory models (GBTM), of which one compared sodium values at 48 hours after ICU admission, and the other two at 72 hours.^(18,25,27)^ Minimal change was described by three authors, in which patients were categorized into a sodium group (normonatraemic, hyponatraemic, and hypernatraemic) and further categorized if their sodium levels changed by an arbitrary value.^(16,17,24)^ Seven authors referred to fluctuation; three compared ICU admission serum sodium value with the maximum magnitude change during ICU stay,^(8,21,22)^ whilst four referred to the maximum minus minimum serum sodium value during ICU stay.^(9,14,19,20)^ Four authors referred to variability, but two used the measured standard deviation,^(7,10)^ whilst two used the measured covariate of variation.^(15,23)^

**Table 3:** Terminologies and definitions of dynamic serum sodium change in the literature

Terminology	Definition(s)/Statistical technique
Trajectory	"Group-based trajectory modeling" where patients were placed into three distinct groups depending on whether serum sodium values were ascending, descending, or "stable" at various timepoints, depending on the publication (Huang et al.,^(18)^ Li et al.,^(27)^ Shen et al.)^(25)^
Change	Once a definition of the normal serum sodium range was established, patients were subcategorized into mild, moderate, and severe hypo- and hypernatraemia groups (Darmon et al.,^(16)^ Grim et al.,^(17)^ Troché et al.)^(24)^
Fluctuation	ICU admission serum sodium was recorded and compared with the absolute maximum change in serum sodium during the ICU stay (Marshall et al.,^(21)^ Eagles et al.,^(8)^ Sakr et al.^(22)^) Highest minus lowest serum sodium values during ICU admission were taken (Bales et al.,^(14)^ Jin et al.,^(19)^ Harada et al.,^(9)^ Labib et al.)^(20)^
Variability	Quantified using measured standard deviation (Harrois et al.^(10)^, Cohen et al.)^(7)^ Quantified using measured coefficient of variation (standard deviation divided by the mean and x 100) (Sen et al.,^(23)^ Chua et al.)^(15)^

ICU - intensive care unit.

### Studies in subarachnoid hemorrhage populations

Dynamic changes in serum sodium amongst the adult ICU population have been mostly studied in patients with SAH; between 2014 and 2016, a total of 7 publications encompassing 2,630 patients were reviewed^(7-9,14,15,19,20)^ (Table 1). Six publications included only patients with SAH caused by confirmed rupture of an intracranial aneurysm on computed tomography or catheter-based angiography, whilst one incorporated data from the Medical Information Mart for Intensive Care IV database and included all patients with "spontaneous" SAH not further specified.^(19)^

There was a dearth of studies examining specific anatomical SAH characteristics in relation to the risk of dynamic changes in serum sodium. One study compared sodium fluctuation rates between ruptured anterior communicating artery aneurysms and other locations and found no significant difference.^(20)^ No study has found an association between the extent of sodium fluctuation and World Federation of Neurosurgical Societies grading, age, or sex. Furthermore, no study has found an association between sodium fluctuations and the mode of aneurysm treatment post-rupture.

Among the articles investigating dynamic changes in serum sodium and SAH, four included delayed cerebral ischemia (DCI) as the primary outcome,^(8,9,14,20)^ three of which found that a greater magnitude of sodium fluctuation was associated with a higher incidence of DCI.^(8,9,14)^ Three studies also reported worse functional outcomes, as measured by the modified Rankin Scale, with greater sodium fluctuation,^(7,14,15)^ and one study found a higher rate of in-hospital mortality if sodium fluctuation was > 8.5mmol/L.^(19)^ This same study that explored sodium fluctuation and mortality found that only the use of vasoactive agents revealed a differential effect of sodium fluctuation in predicting in-hospital mortality (p value = 0.033); patients without vasoactive agent use had a worse prognosis with sodium fluctuation above 8.5mmol/L.^(19)^

### Studies in other intensive care unit populations

From the ten identified studies published between 2013 and 2024 focused on non-SAH cohort populations, a total of 80,860 patients have been included^(10,17,18,21,23-27)^ (Table 2). Mortality has been the primary endpoint measure for all ten publications.^(10,17,18,21,23-27)^

Harrois et al. studied sodium fluctuations in severe TBI across 14 ICUs. They concluded that daily serum sodium variability was an independent predictor of 28-day mortality, even after adjustment for TBI severity, diabetes insipidus, osmotherapy, hyponatremia, and hypernatremia.^(10)^ It was also found that sodium variability was highest during the first two days after admission and progressively decreased thereafter.^(10)^ Both mannitol and hypertonic saline (HTS) affected the daily standard deviation of serum sodium, but in different ways; HTS was associated with the onset of hypernatremia, an increased daily mean serum sodium, and daily maximum serum sodium, while mannitol was associated with the onset of hyponatremia and with more frequent serum sodium fluctuations.^(10)^ It was noted that two centers exclusively used mannitol, three centers exclusively used HTS, and nine centers used both agents.^(10)^

In the setting of burns, Sen et al. found that amongst 212 patients in one ICU, after adjusting for total body surface area, age, ventilator days, and ICU stay, a higher coefficient of variation of serum sodium measurements was associated with mortality (odds ratio [OR] 5.8; 95% confidence interval [95%CI] 1.5 to 2.2).^(23)^ Further, a larger variation in sodium ranges in the first ten days of admission may be associated with increased mortality (OR 1.35; 95%CI 1.06 to 1.70).^(23)^

Li et al. retrospectively investigated 514 patients with sepsis-related lactic acidosis at a single US hospital. They found that those with stable, normal fluctuations in serum sodium levels had better 30-day survival rates.^(27)^ The authors incorporated a GBTM and developed Kaplan-Meier curves to model survival amongst patients on differing serum sodium developmental trajectories.^(27)^ However, subgroup analysis uncovered statistically significant interactions (p < 0.05) between different serum sodium trajectories and covariates such as race, marital status, Glasgow Coma Scale, Sequential Organ Failure Assessment, need for renal replacement therapy (RRT), congestive heart failure, kidney disease, liver disease, and diabetes.^(27)^ Shen et al. also retrospectively studied 1,038 cases with sepsis after cardiopulmonary bypass surgery from the same United States hospital and incorporated a similar GBTM.^(25)^ The authors concluded that although the fluctuation of serum sodium remained within the normal range after cardiac surgery (defined as 138 - 141mEq/L), a higher concentration of serum sodium trajectory substantially increased the likelihood of 30-day mortality risk in this cohort.^(25)^

Two studies have explored sodium fluctuations in patients with primary surgical diagnoses requiring ICU; Marshall et al. and Sakr et al. both found an independent association with increased 28-day and in-hospital mortality, respectively, and higher sodium fluctuation magnitude.^(21,22)^ Both studies also found that the association between mortality and sodium fluctuation was present amongst patients who remained normonatraemic throughout their ICU length of stay.^(21,22)^ The study by Marshall et al. included 8,600 patients from multiple ICUs and found that the association between mortality and the magnitude of sodium fluctuation remained even after multivariate adjustment for demographics, illness severity, and comorbidities.^(21)^ Sakr et al. incorporated 10,923 patients from a single ICU, subcategorized them into cardiothoracic, digestive, neurosurgery, trauma, and other patients (47.2%, 24.3% and 16.0%, 4.2% and 8.4%, respectively), but did not delineate whether higher incidences of sodium fluctuations occurred in certain categories.^(22)^

Huang et al. and Troché et al. investigated patients with AKI requiring renal dialysis.^(18,24)^ Huang et al. investigated 9,314 patients and categorized them into distinct sodium trajectory groups as either ‘stable’, ‘descending’, or ‘ascending’.^(18)^ It was found that the "ascending" group had a 16.6% associated 30-day mortality compared with the "stable" and "descending" groups (7.9% and 9.5%, p < 0.001).^(18)^ The results were similar at one-year mortality (p < 0.001), and no significant differences were appreciated for age or gender distribution between the groups.^(18)^ Troché et al. studied 252 critically ill patients. They found a median increase in sodium from 135mM before dialysis to 140mM after, with a median increased rate of one mM per hour.^(24)^ Multivariate analysis found the only significant predictor of sodium variation was the dialysis solution sodium gradient. Mortality increased with the magnitude of increased sodium variability.^(24)^

Among unselected critically ill adult patient populations, two studies have been conducted by Darmon et al.^(26)^ and Grim et al.^(17)^ Darmon et al. investigated 11,125 critically ill patients and found that one-third had mild to moderate dysnatremia at ICU admission.^(26)^ Dysnatremia, including mild changes in serum sodium concentration, was an independent risk factor for hospital mortality.^(26)^ Grim et al. studied 36,660 critically ill patients and found that an increase in serum sodium in the first 48 hours of ICU admission was associated with higher in-hospital mortality in both patients admitted with normonatremia and hypernatremia.^(17)^

## DISCUSSION

To the best of our knowledge, this is the first scoping review exploring the evidence level for clinical outcomes associated with dynamic changes in serum sodium in the setting of adult critical illness. Authors have employed various methods of measuring dynamic changes in serum sodium and consistently reported increased morbidity or mortality amongst patients with spontaneous SAH, surgical patients, TBI, burns patients, acute kidney injury, and sepsis.^(7-10,14-25,27)^ However, these studies were all observational, heterogeneous in design and statistical analysis methods, confounded, and realistically had limited capacity to explore causal mechanisms.

Dynamic changes in serum sodium amongst the adult ICU population have been mostly studied in patients with spontaneous SAH,^(7-9,14,15,19,20)^ reflecting practice in which monitoring serum sodium is a pivotal aspect of basic ICU management of patients with SAH.^(28)^ Both high serum sodium concentrations^(29,30)^ and now greater dynamic changes in serum sodium have been associated with worse functional outcomes and mortality in SAH.^(7-9,14,15,19,20)^ However, the strength of this association would be much improved with a prospective, controlled study design to better guide future ICU SAH management.

Hypernatremia may reflect the severity of brain injury resulting in diabetes insipidus or may be a marker of treatment with hyperosmolar therapy for cerebral edema, both of which are plausible explanations for worse 6-month functional outcomes.^(31)^ Similarly, Cohen et al. have postulated that increased variability may reflect the consequence of attempts to correct hypernatremia with hypotonic fluids or attempts to correct hyponatremia with hypertonic solutions.^(7)^ Contrastingly, the prognostic value of hyponatremia in the setting of spontaneous SAH is mixed; some report that hyponatremia is associated with a greater risk of developing DCI in the setting of SAH,^(32-34)^ whilst others have found no relationship between hyponatremia and functional outcome or mortality.^(29,35)^ Explanations for these literature disparities have included varied definitions of hyponatremia, reporting methods that may not account for exposure duration and hyponatremia severity, and differences in follow-up duration and method.^(7,36,37)^

Dysnatremia is most frequently reported in neurocritical care patients^(10,30)^ compared to unselected critically ill populations^(38,39)^ and this is potentially due to the CNS-mediated causal mechanisms as well as the resultant impacts of serum sodium changes and direct secondary neuronal injury.^(30,40)^ Risk factors for dysnatremia have included hypothalamic injury leading to diabetes insipidus, syndrome of inappropriate antidiuretic hormone, cerebral salt wasting, Addisonian crisis, desmopressin administration, and sodium-based osmotherapy for both treating hyponatremia and critically raised intracranial pressure.^(10)^ But dynamic changes in serum sodium have also been studied in mixed ICU,^(16-18,24)^ surgical ICU,^(21,22)^ sepsis,^(25,27)^ TBI,^(10)^ and burns patient populations.^(23)^ From a total of ten publications, four used all-cause in-hospital mortality^(17,22-24)^ as the primary outcome, four used 30-day mortality,^(16,18,25,27)^ and 2 used 28-day mortality.^(10,21)^ These studies have generally involved larger cohorts than those in SAH populations, providing greater generalisability and underpinning the clinical relevance of dynamic serum sodium changes across all adult critical illnesses.

The greatest extent of serum sodium variation occurred within the first 48 hours of ICU admission, across general ICU,^(18)^ severe TBI,^(10,30)^ and burn patients.^(23)^ In the setting of TBI, Harrois et al. proposed that drastic sodium fluctuations can occur with repeated mannitol or HTS dosing in patients with critically elevated intracranial pressure, reflecting a patient with a catastrophic TBI and an inherently increased mortality risk.^(10)^ Similarly, Sen et al. concluded that dysnatremia and higher sodium variation occur in the setting of greater total body surface area burns and secondary sepsis, both of which independently increase mortality risk.^(23)^ One can postulate that dynamic change in serum sodium may reflect changing volaemic status and end-organ perfusion, but what is known is that conventional fluid balance assessments in the ICU setting remain inadequate.^(41)^

Appropriate techniques for handling a time-varying covariate, such as serum sodium concentration, are essential when evaluating the causal effect of dysnatremia on patient outcomes. The available literature is largely silent on this, with most analyses failing to account for the time-varying nature of serum sodium. Harrois et al. adjusted for the occurrence of daily hyponatremia and hypernatremia, aiming to investigate whether daily fluctuations in serum sodium, as quantified by daily serum sodium standard deviation, are associated with outcome regardless of the level of the serum sodium level they fluctuate around.^(10)^ In contrast, Marshall et al.,^(21)^ Sakr et al.^(22)^ and Jin et al.^(19)^ conducted a multivariate logistic regression model. They included many variables, including Simplified Acute Physiology II Score, which accounts for serum sodium on ICU admission only. But Sakr et al. further assessed the impact of fluctuations in serum sodium levels on outcome by constructing a second logistic regression model comparing incidence of hospital mortality between patients who were normonatraemic throughout ICU stay and those who experienced dysnatremia.^(22)^

Jin et al. argue that excessive correction of serum sodium concentration plays a critical role in the pathogenesis of devastating neurological consequences, such as osmotic demyelination syndrome, suggesting that sodium variability may be more important than the absolute sodium level in SAH and potentially in other critical illnesses.^(19,42)^ Previous studies have shown that both fluid restriction and fluid overload are risk factors for DCI and poor outcome in SAH,^(34,43,44)^ reiterating the complex nature of the problem. Further, Vergouw et al. have shown that, in a patients with SAH, a significant reduction in fluid input was possible while maintaining adequate cardiac preload and thus cerebral blood flow, again supporting the notion that current approaches to fluid, and by extension, serum sodium, in the critical illness setting warrant further examination.^(44)^

When considering sodium and water handling in the ICU population, emphasis is often given to the effects of osmotherapy, mineralocorticoids, and the secondary effects caused by acute CNS or renal pathology.^(2)^ But one must also recognize that a significant intravascular sodium load from drugs using sodium-based diluents (i.e., antimicrobials) in addition to bolus and maintenance sodium-based crystalloid fluids is frequently administered in the ICU.^(45,46)^ The amount of sodium is dependent on the drug and diluent, but up to 30% of a patient's total daily fluid intake can be secondary to saline-containing intravenous drug infusions, potentially adding over 100mmol of sodium per day.^(47)^ As such, some ICUs are changing drug diluents from saline to 5% dextrose in water to reduce this sodium burden.^(47)^ Later during the deresuscitation phase of ICU stay, the effects from the liberal use of loop diuretic therapy, occasionally augmented by adjunctive secondary diuretics (e.g., thiazide, spironolactone, acetazolamide) must also be considered.^(48,49)^

Chloride, the predominant extracellular anion, also warrants careful consideration, given that most intravenously administered sodium is in the form of sodium chloride, and hyperchloraemia is associated with renal impairment and a higher RRT requirement.^(50)^ This is also particularly relevant to sodium handling, as again serum chloride concentration may impact inherent kidney-driven natriuresis mechanisms.^(50)^ As such, future investigations should combine optimal sodium monitoring, investigating dynamic changes as well as absolute values, together with the same variables for chloride. Further, these future studies should also account for chronic pathologies that can concurrently impact sodium handling either through secondary hyperaldosteronism (e.g., cardiac failure, cirrhosis) or long-term impaired natriuresis from chronic renal impairment.^(2)^

Glucose, another key determinant of serum osmolarity besides sodium, has been repeatedly investigated in the ICU population, and hyperglycemia has been associated with poor outcomes in several critical illness processes.^(51,52)^ Glycemia variability during ICU stay, measured by the coefficient of variation, has also been explored in ICU sepsis and AKI populations, with higher glycemia variability associated with higher mortality, independent of the mean glucose value.^(53,54)^ This notion that dynamic changes in many ICU-recorded variables are more indicative of disease trajectory or ICU-level care than absolute abnormal values warrants further research. Especially given that cells, tissues, and organs can exhibit a myriad of adaptation mechanisms, studies of absolute values may capture only an incomplete picture.

Another important concept, although beyond the scope of this review, is the cumulative exposure dose, or ‘area under the curve’, separate from dynamic changes, using ICU-captured variables. Modelling this can be inherently challenging, partly due to the need for high-volume, robust data and the presence of many confounders. However, it can be appreciated that serial exposures are likely to be more damaging than an isolated event.^(55,56)^ This concept has so far been particularly shown well in the realm of intracranial pressure "dose" amongst cohorts of TBI and SAH.^(55,56)^

Strengths of this exploratory scoping review included the use of five well-established medical literature databases, inclusion of grey literature, and a thorough search using a comprehensive search strategy designed with the assistance of an expert medical librarian. Limitations include high heterogeneity among studies, which limit systematic analysis, as well as an inability to fully capture the complex temporal evolution of dysnatremia during critical illness (both due to disease and treatment response). Other considerations include the high likelihood of residual confounding in observational studies and the notion that dysnatremia is merely a risk marker rather than a causal agent.

## CONCLUSION

The current literature supports the notion that greater dynamic changes in serum sodium across a range of adult critical illnesses can be detrimental. However, thus far, the associations have only been reported from observational studies and mostly without correction for time-varying covariates. Further research is required to assess the causal relationship between dynamic changes in serum sodium and outcomes, and to determine whether specific serum sodium-targeted practice can improve patient-centered outcomes in the intensive care unit.

## Data Availability

Data will be made available to readers upon request at the discretion of the senior author.
