# Supplementary material for: Clinical outcomes associated with dynamic changes in serum sodium amongst adult patients with spontaneous subarachnoid hemorrhage and other critical illnesses: an exploratory scoping review
Source: Crit Care Sci. 2026 Jul 7;38:e20260019. doi: 10.62675/2965-2774.20260019 (PMC13399389; doi:10.62675/2965-2774.20260019)
Supplement: Supplementary Material [file 2965-2774-ccsci-38-e20260019-Suppl01.pdf]

Critical Care Science
https://doi.org/10.62675/2965-2774.20260019

SUPPLEMENTARY MATERIAL

Clinical outcomes associated with dynamic changes in serum sodium amongst adult patients with spontaneous subarachnoid hemorrhage and other critical illnesses: an exploratory scoping review

Vignesh Raman[1], Mahesh Ramanan[1], Felicity Edwards[1], Zemedu Aweke Ferede[1], Vivienne Tippett[1], Kevin B. Laupland[1]

Table 1S - Search strategy

Database	Search	Search sate	Filter	Hits
PubMed®	(((“Intensive Care Units”[Mesh]) OR (intensive[Title/Abstract] OR critical[Title/Abstract] OR ICU[Title/Abstract])) AND ((sodium[Title/Abstract]) OR ((“Sodium” [Mesh])))) AND ((dysnatraemia[Title/Abstract] OR dysnatremia[Title/Abstract] OR hyponatraemia[Title/Abstract] OR hyponatremia[Title/Abstract] OR hypernatraemia[Title/Abstract] OR hypernatremia[Title/Abstract]) OR (“Hyponatremia”[Mesh]))	30/3/2025	English<br>Humans	319
Embase	(‘intensive care’/de OR intensive:ti,ab OR icu:ti,ab OR critical:ti,ab) AND (dysnatraemia OR ‘dysnatremia’/exp OR ‘hyponatremia’/exp OR ‘hypernatremia’/exp OR hypernatr*:ti,ab OR dysnatr*:ti,ab OR hyponatr*:ti,ab) AND (‘sodium’/exp OR ‘na’:ti,ab OR ‘natrium’:ti,ab OR ‘sodium’:ti,ab) AND [embase]/lim NOT ([embase]/lim AND [medline]/lim) AND [humans]/lim AND [english]/lim)	30/3/2025	Human<br>English	660
CINAHL	((MH “Intensive Care Units+”) OR AB (intensive OR critical OR ICU) OR TI (intensive OR critical OR ICU)) AND ((MH “Hyponatremia”) OR TI (hyponatr* OR hypernatr* OR dysnatr*) OR AB (hyponatr* OR hypernatr* OR dysnatr*) OR ((MH “Sodium”) OR (TI sodium OR AB sodium))	30/3/2025	English	421
Scopus	((ALL (intensive AND care AND unit) OR TITLE-ABS (intensive) OR TITLE-ABS (icu) OR TITLE-ABS (critical)) AND (ALL (hyponatremia) OR TITLE-ABS (hyponatremia) OR TITLE-ABS (hyponatraemia) TITLE-ABS (hypernatremia) OR TITLE-ABS (hypernatraemia) OR TITLE-ABS (dysnatremia) OR TITLE-ABS (dysnatraemia) OR TITLE-ABS (sodium)) AND (LIMIT-TO (LANGUAGE , “English”)) AND (LIMIT-TO (EXACTKEYWORD , “Human”))	30/3/2025	Human<br>English	1,564
Web of Science	(ALL=(intensive care unit) OR TI=(intensive OR critical OR ICU) OR AB=(intensive OR critical OR ICU)) AND (ALL=(hyponatremia) OR ALL=(hypernatremia) OR ALL=(dysnatremias) OR TI=(hypernatremia OR hypernatraemia OR hyponatremia OR hyponatraemia AND sodium) OR AB=(hypernatremia OR hypernatraemia OR hyponatremia OR hyponatraemia AND sodium))	30/3/2025	English	1,363

This is an open access article under the CC BY license (https://creativecommons.org/licenses/by/4.0/).
